# Effects of Multimicronutrient Supplementation during Pregnancy on Postnatal Growth of Children under 5 Years of Age: A Meta-Analysis of Randomized Controlled Trials

**DOI:** 10.1371/journal.pone.0088496

**Published:** 2014-02-20

**Authors:** Wei-Ping Lu, Min-Shan Lu, Zong-Hua Li, Cai-Xia Zhang

**Affiliations:** Department of Medical Statistics and Epidemiology, School of Public Health, Sun Yat-sen University, Guangzhou, People’s Republic of China; Iran University of Medical Sciences, Iran (Republic of Islamic)

## Abstract

**Background:**

The beneficial effect of antenatal multiple micronutrients supplementation on infant birth outcomes has been proposed by previous meta-analyses. However, their benefits on postnatal health of children have not been summarized. A meta-analysis of randomized controlled trials was conducted to evaluate the effect of maternal multimicronutrient supplementation on postnatal growth of children under 5 years of age.

**Methods:**

We searched both published and ongoing trials through the PubMed, EMBASE, CENTRAL (OVID platform), Web of Science, BIOSIS Previews, Chinese Science Citation Database, Scopus, ProQuest, ClinicalTrials.gov, Chinese Biomedical Database, and WANFANG database for randomized controlled trials. Reference lists of included studies and relevant reviews were also reviewed for eligible studies. Standard mean difference (SMD) was employed as the index for continuous variables by using fixed effects models. Trend analysis by visual inspection was applied to evaluate the change of mean difference of weight and height between the groups over time.

**Results:**

Nine trials (12 titles) from nine different countries were retrieved for analysis. Pooled results showed that antenatal multimicronutrient supplementation increased child head circumference (SMD = 0.08, 95% CI: 0.00–0.15) compared with supplementation with two micronutrient or less. No evidence was found for the benefits of antenatal multimicronutrient supplementation on weight (*P* = 0.11), height (*P* = 0.66), weight-for-age z scores (WAZ) (*P* = 0.34), height-for-age z scores (HAZ) (*P* = 0.81) and weight-for-height z scores (WHZ) (*P* = 0.22). A positive effect was found on chest circumference based on two included studies.

**Conclusions:**

Antenatal multimicronutrient supplementation has a significant positive effect on head circumference of children under 5 years. No impact of the supplementation was found on weight, height, WAZ, HAZ and WHZ.

## Introduction

Maternal micronutrients deficiency during pregnancy is an outstanding public health issue worldwide. Due to the increased nutritional requirement, pregnant women are vulnerable to micronutrients deficiency. It was estimated that 41.8% of women during pregnancy suffer from anemia globally [1]. Vitamin A deficiency is another important issue, especially in middle and low-income countries. According to the estimation by WHO, 122 countries were affected by Vitamin A deficiency during the period between 1995 and 2005 [2]. Compared with Vitamin A, Vitamin D deficiency is more universal. The issue is not restricted to developing countries, but also, it is quite common in the industrialized world. In Belgian, it was reported that 74.1% of pregnant women were vitamin D deficiency [3].

Maternal micronutrients insufficiency correlates significantly with the adverse outcomes of their offspring. Iron deficiency anemia of pregnant women were associated with low birth weight (LBW), preterm birth and intrauterine growth restriction (IUGR) [4], [5]. Micronutrients deficiency during pregnancy not only leads to inferior pregnant outcomes, but also has long term and even intergenerational impact on offspring. Preterm, IUGR and small for gestational age infants are inclined to experience growth retardation in their later life [6], [7].

Micronutrients supplementation was considered to be a potential cost-effective and practical strategy to combat the global nutritional challenge of children and women [8], [9]. However, the beneficial effect of antenatal micronutrients supplementation on postnatal growth of children is still unclear. It was reported that children of mothers with higher levels of vitamin C during pregnancy may be taller and heavier at the age of 6 and 12 months [10]. In another study carried out in Indonesia, vitamin A supplementation among pregnant women improves child growth at certain periods in the first two years compared with the combined supplementation of zinc and vitamin A [11]. In one study in Bangladesh, no effect of prenatal zinc supplementation exists on child growth at 6 months [12]. While in another study conducted in Bangladesh, maternal zinc supplementation during pregnancy impedes the growth of their children at the age of 6 and 13 months [13].

Combining multiple micronutrients in a single delivery mechanism has been suggested as a cost-effective way to achieve multiple benefits [14]. The average birth weight of children in the multimicronutrient supplementation group was heavier than that of the children in the iron+folic group in the trial from Iran, the difference did not persist postnatally [15]. However, Schmidt *et al.* [16] failed to observe any differences of height gain between multimicronutrient and control groups during the first year of infancy. By contrast, children in the multimicronutrient group seemed to grow faster than their counterpart in the control group in the study by Zekavat *et al,* though the net height did not differ significantly [15]. A recent meta-analysis concluded that multiple micronutrients supplementation during pregnancy contributes to lower risk for LBW and small-for-gestational age (SGA) [17]. This result was consistent with a meta-analysis reported by Haider *et al.* [18]. However, the supplementation of multimicronutrients failed to generate other maternal and infant benefits like preterm birth, stillbirth, maternal or neonatal mortality [17], [18].

To our knowledge, findings of the effect of antenatal micronutrients supplementation on child growth were still unclear, and these data have not been systematically evaluated. Therefore, we conducted a meta-analysis of randomized controlled trials (RCTs) to quantitatively evaluate the beneficial effects of multiple micronutrient supplements during pregnancy on postnatal child growth.

## Materials and Methods

This meta-analysis was performed according to the recently published recommendations and checklist of the Preferred Reporting Items for Systematic Reviews and Meta-Analyses (PRISMA) statement.

### Search Strategy

We searched for all published articles indexed in PubMed, EMBASE, CENTRAL (OVID platform), Web of Science, BIOSIS Previews, Chinese Science Citation Database, Scopus, ProQuest, ClinicalTrials.gov, Chinese Biomedical Database and WANFANG database. The time frame was limited to between January 1990 and May 18, 2013 and the language was limited to English and Chinese. The search strategies used the following key words: (1) micro-nutrient* OR micronutrient* OR multimicronutrien* OR multi-micronutrien* OR multi-nutrient* OR multinutrient* OR vitamin* OR multi-vitamin* OR multivitamin* OR “trace element*” OR iron OR ferrous OR ferric OR “folic acid” OR folate* OR folvite OR folacin* OR pteroylglutamic OR zinc OR calcium OR selenium OR mineral* OR multimineral* OR multi-mineral* OR iodine OR thiamine OR riboflavin* OR magnesium OR carotene* OR copper OR niacin*; (2) pregnan* OR maternal OR mother OR antennal OR ante-natal OR prenatal OR pre-natal OR female*; (3) child* OR infan* OR baby OR babies OR pre-school* OR preschool* OR postnatal OR post-natal; (4) grow* OR anthropometr* OR weigh* OR height OR length; (5) (1) AND (2) AND (3) AND (3) AND (4). We also manually searched the reference lists of every primary study for additional publications. Further searches were done by reviewing review articles.

### Inclusion and Exclusion Criteria

Only randomized controlled trials (RCT) that address effects of maternal multiple micronutrients supplementation during pregnancy on the growth of children under 5 years were eligible for inclusion. Studies comparing the outcomes of supplementing pregnant women with multiple micronutrient supplements of three or more micronutrients compared with placebo, or no supplementation, or supplementation with two or less micronutrients.

The exclusion criteria were as follows: (1) HIV positive pregnant women, (2) studies that focused on the growth of children over 5 years, (3) pregnant women in the intervention group were supplemented with only two or less micronutrients, (4) pregnant women were supplemented with more than two micronutrients in control group, and (5) no full text or eligible information was available.

### Data Extraction and Quality Assessment

Data extraction form from the Cochrane group [19] was adapted for data and information abstraction. The main contents extracted from the original study included general information of the studies, participants and their settings, intervention and methodology descriptions, bias assessments, baseline characteristics of the pregnant women, results of the studies and key conclusions from the author of the original studies.

The bias of the included studies was assessed against the criteria provided by Cochrane group in *the Cochrane Handbook for Systematic Reviews of Interventions* [20]. Briefly, seven items were evaluated, *i.e.* random sequence generation, allocation concealment, blinding of participants and personnel, blinding of outcome assessment, incomplete outcome data, selective outcome reporting, and other biases. Accordingly, each item was classified into high, low or unclear risk.

### Statistical Analysis

Primary outcomes were weight and height of children under five years. The secondary outcomes comprised head circumference (HC), chest circumference (CC), weight-for-age z score (WAZ), height-for-age z score (HAZ) and weight-for-age z score (WHZ). Standard mean difference (SMD) was employed as the effect size of continuous variables. It was calculated by dividing mean difference between intervention group and control group by the pooled standard deviation (SD). On occasions that only the standard error (SE) was given, SD was calculated by multiplying the SE with the square root of the sample size. During the follow-up period, the anthropometric measures of the children might be assessed more than once; the result of the last measurement was utilized to assess the outcome of interest [21]. When more than one intervention or control groups coexist in a single study, we first choose the pregnant women who were supplemented with the largest combinations of micronutrients and did not receive any other treatment as the intervention group; pregnant women supplemented with two or less micronutrient or none were taken as the control group. Then, we used the pooled means of the intervention or control groups defined by the original study design for sensitivity analysis. In the Maternal and Infant Nutrition Interventions in Matlab (MINIMat study) [22], [23], the participants were divided into two different groups who received early food and usual food supplementation, respectively. In each of the group, pregnant women were further divided into three subgroups. Participants in the three subgroups were supplemented with multiple micronutrients or 60 mg iron+folic acid or 30 mg iron+folic acid. The two groups were treated as two different studies for analysis.

We also attempted to investigate the trend of the mean difference of the outcomes between the intervention and control groups over time by visual inspection. For dichotomous variables, the pooled Mantel-Haenszel risk ratio and its 95% confidence interval (95% CI) were reported. We did a *post hoc* model selection for data synthesis. Heterogeneity was assessed by using *T* ^2^, *I* ^2^ and *Chi* ^2^ statistics, significant heterogeneity was confirmed when *I* ^2^>50% or *P*<0.1. We presented the results on the basis of a random-effect model when the test for heterogeneity was significant. Otherwise, the results of a fixed-effect model were presented. We also used funnel plot asymmetry to detect any potential publication bias and Egger’s regression test to measure funnel plot asymmetry. The between-study data synthesis and heterogeneity assessment were performed with RevMan software (version 5.1; The Cochrane Collaboration, Oxford, United kingdom) [19]. Comprehensive meta-analysis (Comprehensive Meta-analysis Version 2, Biostat, Englewood NJ) was employed to pool within-study data (when there are multiple intervention or control groups) and to perform the trend analysis. STATA (Version 12.0, Stata Corp, College Station, Texas) was used to perform Egger’s regression test.

To explore the possible influence of those covariates on child growth change, we further conducted a series of pre-specified subgroup analyses stratified by duration of the intervention (< = 25 weeks vs. >25 weeks), supplemental differences [United Nations International Multiple Micronutrient Preparation (UNIMMAP) *vs.* Non-UNIMMAP] and age of children (< = 12 months vs. >12 months). For studies of high risk, sensitivity analysis were undertaken to explore risk factors that have an impact on the outcomes and to assess the robustness of the results.

## Results

### Study Identification

**Figure 1:**
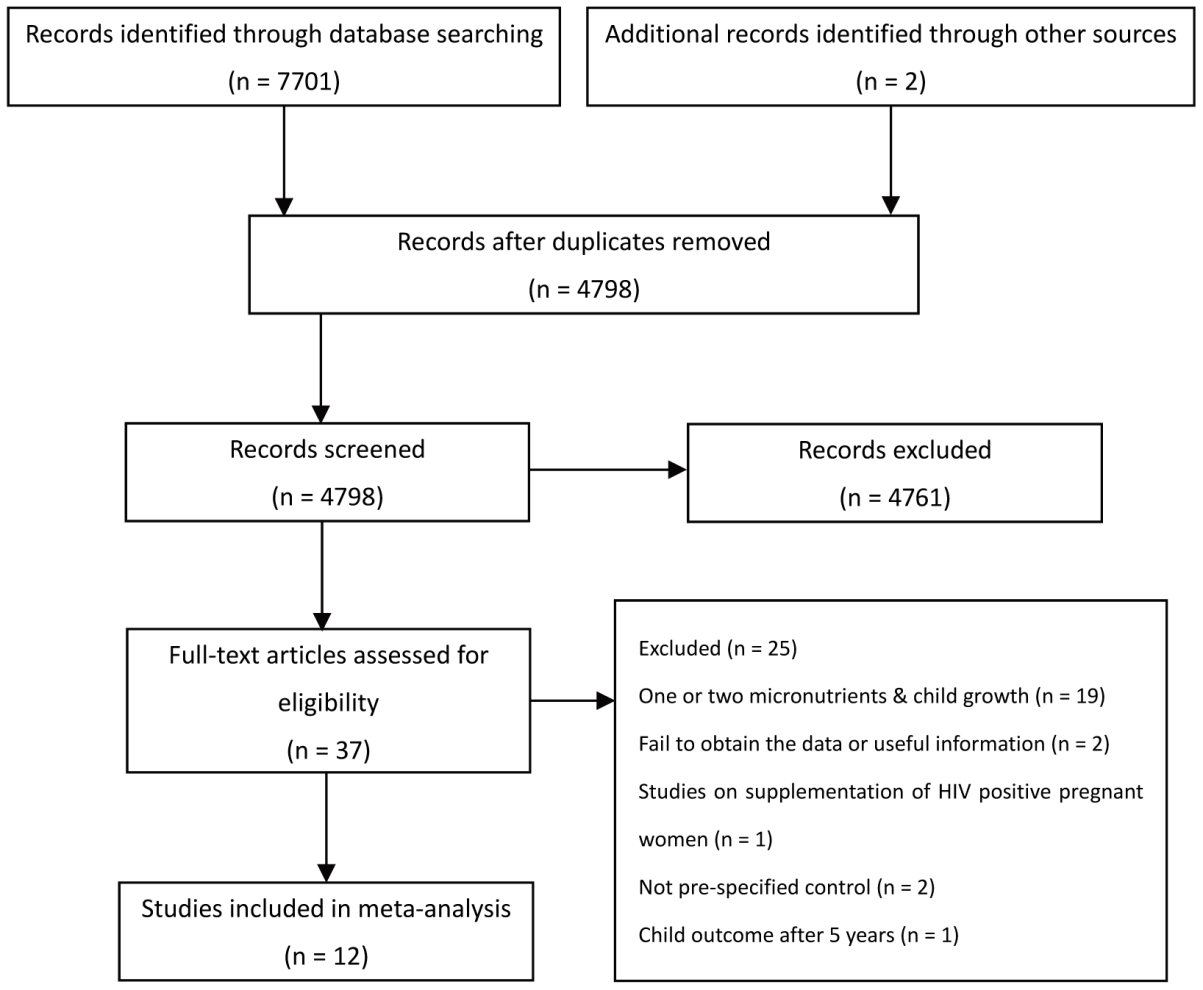
Selection of studies included in meta-analysis on antenatal multi-micronutrient supplementations on postnatal child growth under 5 years.

The detailed processes of the study selection are shown in **Figure 1**. We identified seven titles in Chinese [24]–[30] and thirty titles in English [11]–[13], [15], [16], [22], [23], [31]–[54] that were potentially eligible. Of the seven Chinese studies, five were about effects of one or two micronutrients supplementation during pregnancy on posterity growth and were excluded [25]–[29]. Two studies explored the effect of maternal multiple micronutrients supplementation on postnatal growth of the children [24], [30]. However, we failed to obtain full text or useable information of the two eligible studies and hence they were excluded. Of the thirty titles in English, one investigated vitamin supplementation of HIV positive pregnant women and the effect on child growth [47], fourteen investigated antenatal supplementation with one or two micronutrients supplementation and were excluded [11]–[13], [39], [41], [42], [44], [45], [48]–[50], [52]–[54]. The remaining fifteen articles examined the effect of maternal supplementation of multiple micronutrients, of which two have no prespecified control groups [40], [43] and the one evaluated the outcomes of children over 5 years old [51]. The remaining twelve articles [15], [16], [22], [23], [31]–[38] were derived from nine different trials and were included for final analysis. We also identified two ongoing trials registered in ClinicalTrial.gov [55], [56]. No data was available from these two studies for analysis.

### Characteristics and Quality of the Included Studies

**Table 1** summarizes the characteristics of the included studies. Of the twelve included titles from nine trials, eleven are full reports [15], [16], [22], [23], [31], [33]–[38], one is a conference abstract [32] that provided eligible data. Nine trials were from eight different countries. Two were from Indonesia [16], [31], [33], the other seven studies were from Mexico [32], Peru [34], [35], Nepal [36], Bangladesh [22], [23], Burkina Faso [37], China [38] and Iran [15], respectively. Five studies [22], [23], [36]–[38] used UNIMMAP package which contains 15 different micronutrients for the intervention group. In most of the studies, the control group received supplement with iron and folic acid or iron alone except one study took placebo as control [15]. Almost all of the included studies initiated the interventions in the second trimester (13–27 gestational weeks) until delivery. Only one study offered the supplements to the enrolled women at 8–10 weeks of gestation [32]. The duration of the interventions ranged from 16 to 32 weeks. After delivery, the minimum follow-up period of newborn infants was three month [32] and the maximum was fifty-four months [22], [23].

**Table 1:** Characteristics of the included studies selected for meta-analysis.

Study ID	Study design	Participant number^1^	Region	Study time^2^	Interventiontime (weeksof gestation)	Duration of intervention (weeks)	Interventiongroup	Control group	Measure time (months of children)^3^	Outcome measurement
Schmidt, 2001	RCT	366 mothers,222 children	Indonesia	1997–1998	18	22	Vitamin A+iron+folicacid	Iron+folic acid	4	Weight, height
Neufeld, 2002	RCT	NG^4^ mothers,460 children	Mexico	NG	8–10	30–32	MMN^5^	Iron	3	Weight, height
Schmidt, 2002	RCT	243 mothers,193 children	Indonesia	1997–1999	18	22	Vitamin A+iron+folicacid	Iron+folic acid	12	Weight, height
Dijkhuizen, 2004	RCT	230 mothers,136 children	Indonesia	1998–1999	16	24	β–Carotene or zinc orboth+iron+folic acid	Iron+folic acid	6	WAZ^6^, HAZ^7^, WHZ^8^
Iannotti, 2007	RCT	1295 mothers,444 children	Peru	1995–1998	16	24	zinc+iron+folic acid	Iron+folic acid	1–12/monthly	Weight, height, head circumference, chest circumference
Iannotti, 2008	RCT	1295 mothers,461 children	Peru	1995–1998	16	24	zinc+iron+folic acid	Iron+folic acid	1–12/monthly	Weight, height, head circumference, chest circumference
Vaidya, 2008	RCT	1200 mothers,917 children	Nepal	2005–2006	16	24	UNIMMAP^9^	Iron+folic acid	30	Weight, height, head circumference, chest circumference, WAZ, HAZ, WHZ
Khan, 2011^10^	RCT	4436 mothers,1634 children	Bangladesh	2001–2009	14	26	UNIMMAP	Fe60F/Fe30F	3,6,18,24,54	WAZ, HAZ, WHZ
Khan, 2012^10^	RCT	4436 mothers,2290 children	Bangladesh	2001–2009	14	26	UNIMMAP	Fe60F/Fe30F	54	Weight, height, head circumference
Roberfroid,2012^11^	RCT	1426 mothers,1169 children	Burkina Faso	2004–2008^12^	24	16	UNIMMAP	Iron+folic acid	1–12/monthly &30 month	WAZ, HAZ, WHZ
Wang, 2012^13^	ClusterRCT	5828 mothers,1388 children	China	2002–2006	14	26	UNIMMAP	Iron+folic acid/folic acid	30	Weight, height, head circumference, WAZ, HAZ, WHZ
Zekavat, 2012^14^	RCT	60 mothers, NG children	Iran	2007–2008	Third trimester	NG	Multivitamins	Zinc/placebo	3	Weight, height, head circumference

1 including number of mothers enrolled and number of children whose anthropometric data was provided;

2 from the enrollment until the final measurement of the children;

3 time points at which growth of child were measured;

4 NG means not given;

5 1–1.5 RDA of several micronutrients, details of the component were not given;

6 WAZ means weight-for-age z scores;

7 HAZ means height-for-age z scores;

8 WHZ means weight-for-height z scores;

9 UNIMMAP contains 15 vitamins and trace elements. They are vitamin A (800 µg), vitamin E (10 mg), vitamin D (5 µg), vitamin B1 (1.4 mg), vitamin B2 (1.4 mg), niacin (18 mg), vitamin B6 (1.9 mg), vitamin B12 (2.6 µg), folic acid (400 µg), vitamin C (70 mg), iron (30 mg), zinc (15 mg), copper (2 mg), selenium (65 µg), and iodine (150 µg);

10 there were six intervention groups including UNIMMAP, Fe60F, Fe30F plus early or usual food invitation. Fe60F means 60 mg iron+folic acid; Fe30F means 30 mg iron+folic acid;

11 the effect of intervention was estimated with mixed effect models;

12 estimated based on the descriptions from the study;

13 the effect of the intervention was estimated with mixed effect linear and logistic model;

14 data was not provided for analysis.

The included trials were of variable methodological quality. Participants were adequately randomized to the treatment groups in five trials [22], [23], [36]–[38] whereas the method used for generating the randomization sequence was not described in sufficient detail in the remaining studies [15], [16], [31]–[35] to permit judgment. Allocation of participants into the intervention and control groups was concealed in nine trials [16], [22], [23], [33]–[38], whereas allocation was unclear in the remaining three trials [15], [31], [32]. In nine trials [15], [22], [23], [33]–[38], the participants, caregivers and the outcome assessors were blinded to the treatment allocation. In other three trials, one [31] showed blinding of participants and caregivers; one [16] showed blinding of outcome assessors only; and the remaining study [32] was a double blind trial but it was not clear as to who was blinded. Loss to follow up was less than 10% in nine trials [16], [22], [23], [31], [34]–[38]. It was more than 10% in one trial [33]; and no reported in two trials [15], [32]. Intention-to-treat analysis was used in all of the trials. As a whole, the risk of the bias of the included studies was relative low except two trials [32], [33].

### Effect on Anthropometry of Children

**Figure 2:**
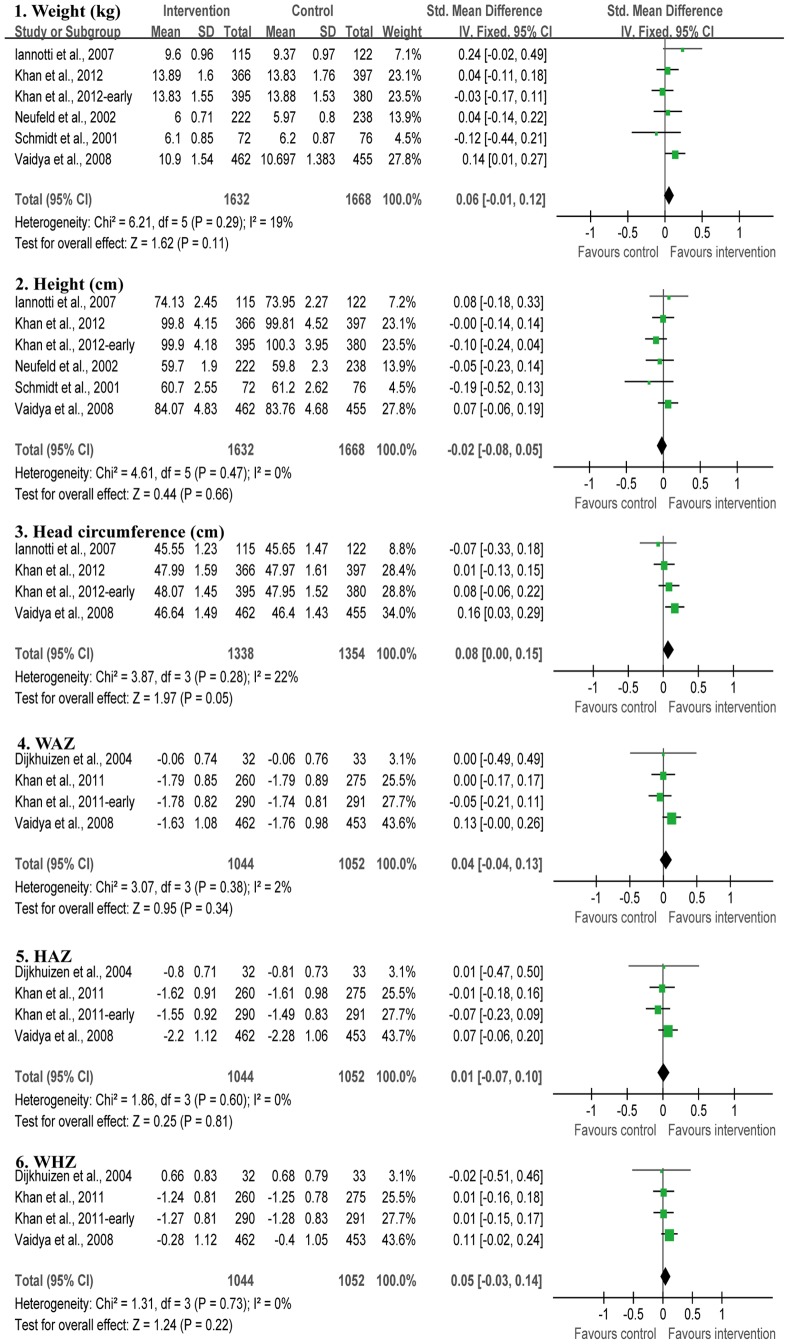
Effect of antenatal multimicronutrient supplementation on child growth under 5 years in randomized controlled trials.

We noted that there were no significant between-study heterogeneity in the effects of antenatal multimicronutrient supplementation on child growth at weight, height, head circumference, chest circumference, WAZ, HAZ and WHZ. Coefficients of inconsistency (*I^2^*) were 19%, 0%, 22%, 0%, 2%, 0% and 0%, respectively. Using the fixed-effects model, antenatal multimicronutrient supplementation increases child head circumference (SMD = 0.08, 95% CI: 0.00–0.15, *p* = 0.05) compared with supplementation with two micronutrient or less. However, antenatal multimicronutrient supplementation did not significantly affect weight (SMD = 0.06, 95% CI: −0.01–0.12, *P* = 0.11), height (SMD = −0.02, 95% CI: −0.08–0.05, *P* = 0.66), WAZ (SMD = 0.04, 95% CI: −0.04–0.13, *P* = 0.34), HAZ (SMD = 0.01, 95% CI: −0.07–0.10, *P* = 0.81) and WHZ (SMD = 0.05, 95% CI: −0.03–0.14, *P* = 0.22) (**Figure 2**). In terms of the effect of multimicronutrient supplementation on chest circumference, a positive effect (SMD = 0.34, 95% CI: 0.08–0.16, *P* = 0.01) was found based on two included studies (data not shown in figures).

### Effect on Growth Trend of Children

**Figure 3:**
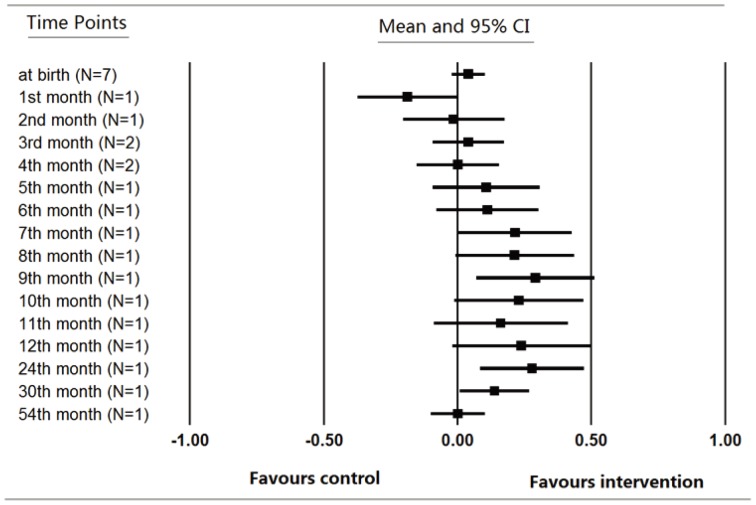
Mean difference of weight over time.

**Figure 4:**
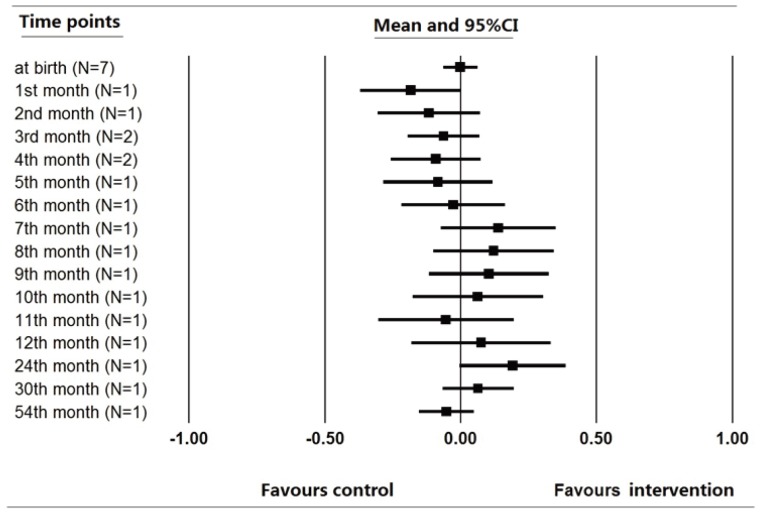
Mean difference of height over time.

Since the growth trajectory of children may change over time, it is possible that the difference of growth measures were not stable in childhood. The trend of the mean difference of the outcomes was explored by visual evaluation. For studies [15], [16], [22], [23], [31]–[38] providing data of weight and height for analysis, we choose weight and height as the measurement for the trend analysis. The pooled mean difference of weight and height at different time points were plotted (**Figure 3** & **Figure 4**). As illustrated in **Figure 3** (*N* stands for the number of studies from which the data was pooled), the mean difference of weight between children in MMN group and control group seemed to increase over time during the first year of infancy gradually and then reach the plateau after that. By contrast, no obvious trend of growth or decrease was observed in **Figure 4**. The height of children did not differ between different groups at any of the observational periods. The trend analysis enhanced the reliability of our findings that MMN supplementation of pregnant women benefit the growth of weight rather than height of children.

### Subgroup and Sensitivity Analyses

In general, there were no statistically significant differences in the pooled effects of antenatal multimicronutrient supplementation on weight, height, WAZ, HAZ and WHZ between varied subgroups stratified by duration of the intervention (< = 25 weeks vs. >25 weeks), supplemental differences (UNIMMAP *vs.* Non-UNIMMAP), and age of children (< = 12 months vs. >12 months). SMD (95% CI) of weight were 0.13 (0.02–0.24) (*p* = 0.02) and 0.01 (−0.08–0.10) (*p* = 0.82) in the < = 25 and >25 weeks of the intervention groups, respectively. Also SMD (95% CI) of head circumference were 0.12 (0.00–0.23) (*p* = 0.05) and 0.05 (−0.05–0.15) (*p* = 0.36) in the < = 25 and >25 weeks of the intervention groups, respectively. Sensitivity analysis showed that the pooled effects were largely similar when analyses limited to high quality studies (**Table 2**).

**Table 2:** Subgroup analysis of the effects of antenatal multimicronutrient supplementation on postnatal child growth.

Subgroup	No. of studies	SMD (95% CI)	p value	I^2^ for between-studyHeterogeneity (%)	*P* for between-study heterogeneity
*All studies*					
Weight	6	0.06 (−0.01,0.49)	0.11	19	0.29
Height	6	−0.02 (−0.08,0.05)	0.66	0	0.47
Head circumference	4	0.08 (0.00,0.15)	0.05	22	0.28
WAZ	4	0.04 (−0.04,0.13)	0.34	2	0.38
HAZ	4	0.01 (−0.07,0.10)	0.81	0	0.60
WHZ	4	0.05 (−0.03,0.14)	0.22	0	0.73
*Duration of the intervention: < = 25 weeks*					
Weight	3	0.13 (0.02,0.24)	0.02	32	0.23
Height	3	0.04 (−0.07,0.15)	0.49	10	0.33
Head circumference	2	0.12 (0.00,0.23)	0.05	62	0.10
WAZ	2	0.12 (−0.01,0.24)	0.07	0	0.62
HAZ	2	0.07 (−0.06,0.19)	0.28	0	0.82
WHZ	2	0.10 (−0.02,0.23)	0.11	0	0.60
*Duration of the intervention: >25 weeks*					
Weight	3	0.01 (−0.08,0.10)	0.82	0	0.75
Height	3	−0.05 (−0.14,0.04)	0.27	0	0.64
Head circumference	2	0.05 (−0.05,0.15)	0.36	0	0.50
WAZ	2	−0.03 (−0.14,0.09)	0.67	0	0.68
HAZ	2	−0.04 (−0.16,0.08)	0.50	0	0.63
WHZ	2	0.01 (−0.10,0.13)	0.84	0	0.99
*Supplements: UNIMMAP*					
Weight	3	0.05 (−0.03,0.13)	0.19	37	0.21
Height	3	−0.01 (−0.09,0.07)	0.86	29	0.25
Head circumference	3	0.09 (0.01,0.17)	0.03	17	0.30
WAZ	3	0.04 (−0.04,0.13)	0.34	34	0.22
HAZ	3	0.01 (−0.08,0.10)	0.81	0	0.39
WHZ	3	0.05 (−0.03,0.14)	0.20	0	0.55
*Supplements: Non-UNIMMAP*					
Weight	3	0.07 (−0.07,0.20)	0.33	34	0.21
Height	3	−0.04 (−0.17,0.10)	0.55	0	0.43
Head circumference	1	−0.07 (−0.33,0.18)	0.57	–	–
WAZ	1	0.00 (−0.49,0.49)	1.00	–	–
HAZ	1	0.01 (−0.47,0.50)	0.96	–	–
WHZ	1	−0.03 (−0.51,0.46)	0.92	–	–
*Age of children: < = 12 months*					
Weight	2	0.00 (−0.16,0.16)	0.98	20	0.28
Height	2	−0.08 (−0.24,0.08)	0.31	0	0.44
Head circumference	0	–	–	–	–
WAZ	1	0.00 (−0.49,0.49)	1.00	–	–
HAZ	1	0.01 (−0.47,0.50)	0.96	–	–
WHZ	1	−0.03 (−0.51,0.46)	0.92	–	–
*Age of children: >12 months*					
Weight	4	0.07 (−0.01,0.14)	0.08	40	0.17
Height	4	−0.00 (−0.08,0.08)	1.00	6	0.36
Head circumference	4	0.08 (0.00,0.15)	0.05	22	0.28
WAZ	3	0.04 (−0.04,0.13)	0.34	34	0.22
HAZ	3	0.01 (−0.08,0.10)	0.81	0	0.39
WHZ	3	0.05 (−0.03,0.14)	0.20	0	0.55
*Limited to high quality study*					
Weight	5	0.06 (−0.01,0.13)	0.12	36	0.19
Height	5	−0.01 (−0.08,0.06)	0.79	11	0.34
Head circumference	4	0.08 (0.00,0.15)	0.05	22	0.28
WAZ	3	0.04 (−0.04,0.13)	0.34	34	0.22
HAZ	3	0.01 (−0.08,0.10)	0.81	0	0.39
WHZ	3	0.06 (−0.03,0.14)	0.20	0	0.55

### Publication Bias

Publication bias was examined by analyzing funnel plots and Egger’s regression tests for all pooled effects. These plots were symmetrical (data not shown), and Egger’s regression also suggested no significant asymmetry of the funnel plot for the overall effects of antenatal multimicronutrient supplementation on weight, height, head circumference, WAZ, HAZ and WHZ (*p* = 0.901, 0.543, 0.276, 0.636, 0.751 and 0.452, respectively) in all eligible studies.

## Discussion

This meta-analysis summarized the effect of multimicronutrient supplementation during pregnancy on postnatal child growth under 5 years old. The results indicated that antenatal multimicronutrient supplementation had a significant positive effect on head circumference of children under 5 years of age. No impact of the supplementation during pregnancy was found on weight, height, WAZ, HAZ and WHZ of children.

Benefits of multiple micronutrients supplementation of pregnant women on birth and pregnant outcomes has been reported by several meta-analyses. Haider *et al* reported that incidence of small-for-gestational age was significant lower in mothers who received multiple micronutrients during pregnancy compared with mothers who were supplemented with iron+folic acid antenatally (Relative Risk = 0.87; 95% CI: 0.81–0.95 (fixed model)) [18]. This was consistent with the meta-analysis by Ramakrishnan *et al* [17]. Additionally, multimicronutrient supplementation during pregnancy also reduced the incidence of low birth weight [17], [18]. However, no evidence was available to indicate whether the benefits on birth outcome will last to postnatal period.

The present meta-analysis focused on the growth of offspring under 5 years of HIV-negative mothers. We found that maternal multimicronutrient supplementation during pregnancy generated positive effects on head circumference of children. Two main sets of explanations could be proposed for such postnatal effects. First, multimicronutrient improves fetal growth, and that gain would be sustained as such during early life. For instance, zinc, contained in the multimicronutrient, is a necessary component for placental alkaline phosphatase and a regulator of insulin-like growth factor I activity in osteoblast formation [57]. Zn supplementation was associated with significantly higher head circumference in a 1995 randomized study of 580 African-American pregnant women [58]. Second, a hypothesis implied that prenatal micronutrients might be required for enzymatic, hormonal, or immunologic pathways that are important for later growth. Prenatal zinc was illustrative of this mechanistic polymorphism. Prenatal zinc may protect the infants from growth faltering episodes of infectious origin [59]. Prenatal zinc might also be important to ensure hyperplasia in muscle cells that will later grow in size [60]. Moreover, greater stores at birth could also trigger the production of more growth hormones, such as insulin-like growth factor I, in early life [61]. Prenatal selenium [62] and vitamin A [63] could also play a role in postnatal growth. However, the statistical significance in the overall estimation may thus be overestimated since the conclusion was drawn from only small number of studies in our analysis. Therefore, more studies are needed to further confirm this conclusion.

Although a positive effect of multiple micronutrients supplementation on chest circumference was also found, only two studies measuring chest circumferences were included in the current analysis [34], [36]. The sum of sample sizes in these two studies may not be sufficiently large to have enough statistical power [64]. Therefore, it was not reliable enough to draw the conclusion that antenatal multimicronutrient supplementation had a significant positive effect on chest circumference. More relevant studies are needed to confirm this association. Meanwhile, no evidence that supported benefits on weight, height, WAZ, HAZ and WHZ were present. The possible explanation for the lack of effects might be that the follow-up time was not long enough to reveal the true effect of maternal multimicronutrient supplementation on children [38].

Subgroup analysis showed a marginally significantly favorable effect on weight and head circumference in the < = 25 weeks of the intervention group but not in the >25 weeks of the intervention group. The inverse relation between weight and head circumference changes and duration of intervention is unclear. It may be related to diet fatigue and thus less attention paid to the diet or lower adherence in prolonged periods of intervention. In terms of height, WAZ, HAZ and WHZ, no significant positive effects were present in both subgroups. Sensitivity analyses showed that the pooled effects were largely similar when analyses limited to high quality studies. It suggested that low quality studies did not overestimate the effect of antenatal multimicronutrient supplementation on child growth.

We also plotted the SMD at different time points for qualitative trend analysis. Since the growth trajectories of children are normally nonlinear, the synthesis of data of earlier and later age is questioned. The interaction of age and treatment was reported in some of the included studies. In the study by Iannotti *et al* [34], the positive effect of intervention diminished over time. Similar result was observed by Roberfroid *et al* [37] who reported that the difference of HAZ of children in intervention and control group narrowed gradually as the children grew up. One intriguing questions arised. That is whether the superiority of weight in childhood in MMN group is just the persistence of difference at birth or is the postnatal accumulation. As reported by Vaidya *et al* [36], the 204 g advantage of adjusted weight of children in intervention group was made up of 77 g existing difference at birth and 127 g postnatal accretion. However, in the study from China, the 42 g difference birth weight of children between the intervention and control group vanished postnatally [38]. Hence, the superiority of birth weight did not guarantee that in later life of children.

### Strengths

In this meta-analysis, only those randomized controlled trials that address effects of maternal multiple micronutrients supplementation during pregnancy on the growth of children under 5 years were included in the overall effect estimation and all sub-analyses. The effects of these studies would represent a long-term steady-state rather than a remodeling transient state. Moreover, all studies, except two small studies, were high-quality trials. Furthermore, we attempted to investigate the trend of the mean difference of weight and height between the intervention and control groups over time by the trend analysis. Finally, the result was unlikely to be due to publication bias. The Egger’s regression tests suggested no significant asymmetry of the funnel plot for all the overall effect estimations in our study.

### Limitations

This meta-analysis had some limitations. First, of 12 included studies, only two studies [22], [23] were followed for 54 months and another for 30 months. The other studies were followed for less than two years. Few long-time follow-up studies limited us to evaluate the long-term effect of multiple micronutrients supplementation during pregnancy on postnatal child health. Second, although SMD has been well widely used as an effect to remove the discrepancies of measure units, the outcome was difficult to interpret [65]. The values of SMD cannot be explained as the magnitude of the treatment effect directly. We did not apply mean difference as our size effect considering that growth conditions at different age and in different settings may vary largely. Third, we only chose the data of the final observation for analysis. For studies with repeated measures, four alternative methods were proposed for analysis *i.e.* the initial or final time-point analysis, specific time-point analysis, all time-points analysis and trend analysis [21]. In this review, we chose to utilize the data of the final observation for analysis due to the insufficiency of data for specific time-point analysis (the pre-specified time points were 3, 6, 12 and 24 months). All time-point analysis was abandoned because this method violates the assumption of independence for statistical inference. Fourth, we failed to investigate the effect of several important confounding factors such as the baseline nutritional status of mothers, social economic status of the family, feeding patterns during infancy because this information provided by our included studies was so limited. Overall, however, the quality of our included studies was methodologically high. The publication bias was not significant of each of our outcomes.

### Conclusions

In conclusion, this meta-analysis showed that antenatal multimicronutrient supplementation had a significant positive effect on head circumference of children under 5 years of age. No impact of the supplementation was found on weight, height, WAZ, HAZ, and WHZ of children. The conclusion needed to be further verified by rigorously designed and implemented clinical trials.

## Supporting Information

Checklist S1 PRISMA 2009 Checklist.
(DOC)
