# Correction: Effects of multimicronutrient supplementation during pregnancy on postnatal growth of children under 5 years of age: a meta-analysis of randomized controlled trials

**DOI:** 10.1371/journal.pone.0096970

**Published:** 2014-04-29

**Authors:** The PLOS ONE Staff

The first author’s name is spelled incorrectly. The correct name is: Wei-Ping Luo.
